# Strategies to modulate the intestinal microbiota and their effects on nutrient utilization, performance, and health of poultry

**DOI:** 10.1186/s40104-018-0310-9

**Published:** 2019-01-15

**Authors:** Sudhir Yadav, Rajesh Jha

**Affiliations:** Department of Human Nutrition, Food and Animal Sciences, College of Tropical Agriculture and Human Resources, University of Hawaii at Manoa, 1955 East-West Rd, Honolulu, HI 96822 USA

**Keywords:** Enzymes, Microbiota, Organic acids, Poultry, Prebiotics, Probiotics

## Abstract

Poultry is widely produced and consumed meat globally. Its demand is expected to continue increasing to meet the animal protein requirement for ever-increasing human population. Thus, the challenge that poultry scientists and industry face are to produce sufficient amount of poultry meat in the most efficient way. In the past, using antibiotics to promote the growth of poultry and manage gut microbiota was a norm. However, due to concerns over potential fatalistic impacts on food animals and indirectly to humans, their use as feed additives are banned or regulated in several jurisdictions. In this changed context, several alternative strategies have been proposed with some success that mimics the functions of antibiotics as growth promoters and modulate gut microbiota for their beneficial roles. These include the use of probiotics, prebiotics, organic acids, and exogenous enzyme, among others. Gut microbiota and their metabolic products improve nutrient digestion, absorption, metabolism, and overall health and growth performance of poultry. This paper reviews the available information on the effect of feed additives used to modulate intestinal microbiota of poultry and their effects on overall health and growth performance. Understanding these functions and interactions will help to develop new dietary and managerial strategies that will ultimately lead to enhanced feed utilization and improved growth performance of poultry. This review will help future researchers and industry to identify alternative feed ingredients having properties like prebiotics, probiotics, organic acids, and exogenous enzymes.

## Introduction

The poultry industry is one of the fastest growing meat producing animal industries. Feed efficiency and high performance of the birds are the crucial goals in poultry production. Also, the quality of diet along with environmental conditions and health of birds need to be considered to achieve these goals. Conventionally, prime poultry feed ingredients are corn and soybean meal (SBM). Despite a rigorous search of alternative feedstuffs, nutritionists have yet not been able to find an alternative that can completely replace corn and SBM, although wheat is included in prominent levels in some parts of the world. There has been remarkable progress in the use of alternative feedstuffs like coproducts, which are typically rich in fiber. Dietary fibers have been found to influence the gut microbial ecology [1]. Feed is possibly the most vital factor in exposing the internal body organs with the external environment via the gastrointestinal tract (GIT). The GIT of poultry is home to a complex and dynamic microbial community [2]. Culture-independent molecular techniques have been used in recent years to characterize microbial diversity and have opened the possibility to study the effect of environmental factors on these microbiota. The principal environmental factor is the diet. The initial studies have revealed ground-breaking results on the interaction of diet with microbiota such as microbial communities shift [3], the energy source for bacteria and selective growth of target bacteria [4]. Gut microbiota interacts within themselves, with their host, and with the diet of the host, whereas commensal bacteria play a pivotal role in host health and metabolism, and pathogenic bacteria cause direct or indirect harmful effects. Thus, feed ingredients should be selected to favor gut condition and maintain a balance between the environment, host, and microbiota. The total number of bacteria in the GIT is higher than the number of eukaryotic cells of the host body. According to Aland and Madec [5], bacteria in the host is divided to three types: dominant bacteria (> 10^6^ CFU/g sample), subdominant bacteria (10^3^ to 10^6^ CFU/g sample) and residual bacteria (< 10^3^ CFU/g sample). The poultry GIT consists of a substantial proportion of Gram-positive, mainly facultative anaerobes from crop to lower ileum, whereas the ceca are composed of *Lactobacillus, Enterococcus,* coliforms, and yeasts [6–8]. In the proventriculus and gizzard, low pH causes a decrease in the bacterial population. In the duodenum, enzymes, high oxygen pressure, and bile salts are responsible for a reduction in microbial concentration whereas, in the lower small intestine and large intestine, the environment is favorable for the growth of diverse microbiota. Oviedo-Rondón et al. [9] defined beneficial gut microbiota having a protective role as the first line of defense against pathogenic bacteria in addition to assistance in specific metabolism and gut structure integrity. Both intestinal and cecal bacterial communities change and were found to diversify with age [10, 11]. Apajalahti et al. [12] reported that the ileum and ceca have a favorable environment for bacterial growth and have as high as 10^9^ to 10^11^ bacteria per gram of content, respectively. The authors found 640 distinct species and 140 bacterial genera in the GIT of poultry, where about 90% of the species are yet to be described.

The GIT bacterial succession starts immediately after hatching and the settlement/colonization of microbiota depends on the egg microbial condition and contamination from hen during laying. Also, the species of bacteria in the GIT is determined during laying depending upon their ability to colonize and their interaction in the GIT [12, 13]. The microbial community (MC) keeps changing throughout maturation of birds and is influenced by several factors including chicken strain, sex, and the rearing environment, within and between individual birds [2]. As the host grows, the microbiota becomes more diverse and tends to be relatively stable in older age. Increased breeding density and thermal stress increase harmful bacteria over beneficial ones [14]. Using environmental factors to modulate the intestinal microbiota is quite irregular and variable to control; instead, gut microbiota populations changes dramatically with the change in composition or density of nutrient as they are potential substrates for bacterial growth.

Diet plays a crucial role in the gut health of host by modulating the GIT bacteria, which can cause either a positive or negative effect on the host, depending on the type of diet [1]. The presence of water-soluble non-starch polysaccharides (WS-NSP) leads to a change in gut microbiota population and diversity. Mathlouthi et al. [15] found an increase in *Lactobacillus* and coliforms along with other facultative bacteria population when the bird’s diet was switched from corn-based to wheat and barley-based. In case of WS-NSP rich diets, the increase in viscosity of digestive content and transit time is noticed along with a higher production of short chain fatty acids (SCFAs) which beneficially regulate ileal motility [16]. Change in gut microbiota with antibiotic supplements in a day-old bird shows an adverse effect on immune system development [17].

It can be noted that the host has multiple ways to control intestinal microbial growth and proliferation. However, the interaction among microbiota and between the microbiota and host mucosa is imperative to maintain gut environment balance. The intervention of dietary factors should consider all these interactions and mechanisms and their relationships with each other. This review discusses the gastrointestinal microbiota in poultry, their positive and negative roles, balance in gut ecology, and different strategies to modulate gut microbiota to improve the health and performance of the poultry.

## The gut microbiota of poultry

The GIT of poultry consists of the esophagus, crop, proventriculus, gizzard, duodenum, jejunum, ileum, cecum, colon, and cloaca. Poultry GIT is much shorter as compared to other mammals relative to their body length. Thus, microbiota that grows in such a small GIT with relatively low transit time requires unique adaptations to adhere to the mucosal wall and to proliferate. The ceca have lower passage rate and are favorable to diverse groups of bacteria, which affect nutrient utilization and overall health of poultry.

### Identification and characterization of microbiota

There are different techniques used to identify and characterize intestinal microbiota such as culture-based, G + C profiling, quantitative PCR, 16S rRNA bases studies, high-throughput sequencing, metagenome shotgun sequencing, and metaproteomic [18]. These microbiota studies started in the 1970s with culture-dependent techniques [19]. Some of the problems with these culture-dependent methods include: only culture selected bacteria out of the diverse digestive microbiota; lack phylogenetically-based classification scheme; unable to detect those present in very low abundance; and bacterial species live in a community and are dependent on one another as well as to the host environment. Therefore, isolating and growing in any selected culture might not be the same as in the host intestinal ecology [12]. To overcome these difficulties and limitations in selective culture, and to identify individual bacteria, the modern approach of examining the microbial DNA extracted from the sample using culture-independent techniques are carried out [2, 18]. Molecular techniques are following the culture method in a contest of increment and diversification of complex microbiota during a different phase of life. These advanced techniques revealed that 90% of the bacteria in the chicken GIT were previously unknown species [18]. Among the molecular techniques, the terminal restriction fragment length polymorphism (TRFLP) was used to compare and contrast microbiota in the duodenum, jejunum, ileum, and ceca [20]. Techniques such as metagenomic shotgun sequencing provide a more in-depth understanding of microbiota functionality in specific environments with strong differentiation between treatments microbiota profile [21]. Similarly, next-generation sequencing has made it possible to determine microbiota dynamics with increased coverage and accuracy [22]. Sequence data are further analyzed by Roche 454-pyrosequencing, Illumina MiSeq, HiSeq, and Ion PGM. Taxonomic assignments were done using QIIME and compare with the public databases like GreenGenes, the ribosomal database project (RDP) and SILVA. Further its followed by functional predictions using PICRUSt and Tax4Fun [23]. Stanley et al. [24] concluded that accurate profiling of microbiota could be done only with a controlled environment, from the day of hatch which essentially determines future microbiota too.

### The composition of gut microbiota

The gastrointestinal tract of poultry, the most extensive body surface exposed to environmental influence, is the home of complex and highly diversified molecularly defined microbiota, containing an enormous number of different species that can be called the microbial community or microbiome. Composition and density of microbiota depend on the microbial composition of the inoculum introduced at hatch, first diet, and host intestinal epithelium [12]. The initial bacteria grow very fast, and the sterile environment soon becomes inhabited by 10^8^ and 10^10^ bacteria per gram of digesta in ileum and cecum, respectively in day 1–3 [12]. The authors also found that the bacterial density reached a maximum in a different section of GIT within the first week of age. In a phylogenetic diversity census study of bacteria in the GIT of chicken, 915 species-equivalents operational taxonomic units (defined at 0.03 phylogenetic distances) were found where chicken sequences represent 117 established bacterial genera [25]. The GIT harbors more than 100 billion bacteria. It consists of several times more bacteria than some of the cell in the host body, including thousands of species dominated by anaerobic bacteria. According to Albazaz and Bal [26], 12 days old birds have around 10–15 times higher facultative and obligatory anaerobic bacteria than that of aerobic bacteria. In a healthy balanced microbial community, there are mostly beneficial gram-positive bacteria (at least 85% of total bacteria), and remaining bacteria includes *Clostridium* in young birds and *Salmonella*, *Campylobacter*, and *E. coli* in older birds without any intestinal disturbance [27]. Some of the commonly found microbes in the GIT of poultry are *Lactobacillus* sp., *Bacteroides* sp., *Eubacterium* sp., *Clostridium* sp., *Escherichia coli, Streptococcus* sp., *Prevotella* sp*.*, *Fusobacterium* sp*.*, *Selenomonas* sp., *Megasphaera* sp., and *Bifidobacterium* sp. Commonly reported cecal microbiota of poultry are summarized in Table 1.

**Table 1:** Presence of dominant microbiota in the ceca of chicken

Dominant microbiota	Reference	No. of genera	Comments
Firmicutes*,* Bacteroidetes, and Proteobacteria (> 90%) *Peptostreptococcus, Propionibacterium, Eubacterium,* *Bacteroides*, and *Clostridium*	Wei et al. [25]	13 phyla and 117 genera	> 900 species-equivalent OTUs, defined at 0.03 phylogenetic distance
*Blautia, Faecalibacterium*, and *Anaerotruncus* (dominant in the reused litter) *Escherichia*/*Shigella, Lactobacillus, Bacteroides*, and *Subdoligranulum *(dominant in the fresh litter)	Wang et al. [98]	133 OTUs within 41 genera considered	Genera differed between the fresh and reused litter for the cecal digesta samples. More abundance in d 10 than d 35
10% previously known species, 35% previously known genus but unknown species, and 55% unknown genus	Apajalahti et al. [12]	> 640 species from 140 genera	Consider bacterial community rather than talking about individual species.
Clostridiaceaen (65%), *Fusobacterium* (14%), *Lactobacillus* (8%) and *Bacteroides* (5%)	Albazaz and Bal [26]		
*Clostridium leptum* (20%), *Clostridium coccoides* (27%), *Sporomusa* sp. (21%), and *Gamma Proteobacteria* groups (20%), *Atopobium* (3.6%), *Bacteroides* (2%), and *Bifidobacteria* (1%)	Zhu et al. [2]		Microbiota from ceca of mature birds fed standard commercial diet
Lachnospiraceae (47%), Ruminococcaceae (19%), *Bifidobacterium* (10%), *Lactobacillus* (10%), Coriobacteriaceae (7%), *Bacteroides* (2%) and others (5%)	Apajalahti and Vienola [43]		Average cecal microbiota composition of commercial broiler chicken farms
Bacteroidetes (> 18%), Tenericutes and Proteobacteria (1%–5%) and at family level Ruminococcaceae, Bacteroidaceae, uncultured Clostridiales, and Streptococcaceae	Witzig et al. [99]		Microbiota present in ceca
*Fusobacterium prausnitzii*, *Ruminococci, Clostridia* and *E. cecorum*	Gong et al. [20]		Present in cecal mucosa

**Fig. 1:**
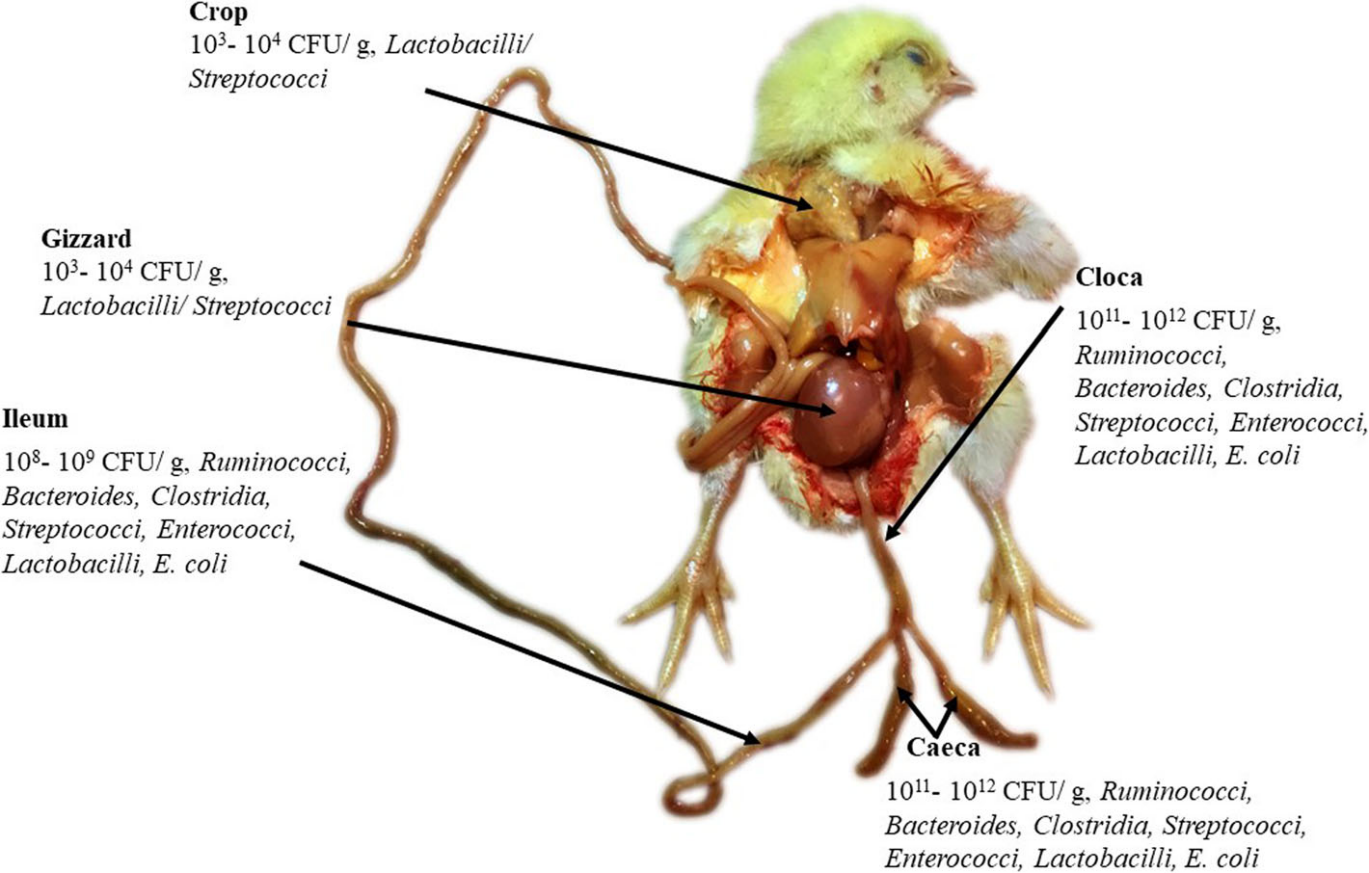
The major bacterial habitats and concentration in the gastrointestinal tract of chicken

The MCs are distributed throughout the GIT of poultry, but due to differences in morphology, functionality, metabolic interactions, and microenvironment, regional heterogeneity in community composition is observed along the different GIT segments [28]. Also, the bacterial concentration gradually increases along the intestinal tract ranging from 10^5^ bacterial cells/g of luminal content in the duodenum to 10^7^–10^12^ bacterial cells/g of luminal content in ileum to the colon, as is illustrated in Fig. 1. According to a recent study on the comparison between the lumen and mucosa-associated bacteria, the mucosa was found to have a highly rich microbial community of distinct group in ileum and cecum [29].

## Role of gut microbiota

Gut microbiota of animals extensively interact with the host, diet, and within themselves [1]. Commensal gut microbiota plays a decisive role in maintaining the normal physiology of host animals. Some of the major roles are to help direct the normal formation or development of gut structure and morphology, boost immune responses, offers protection from luminal pathogens, as well as play an active role in digestion and utilization of nutrients [30]. Gut microbiota also has some direct and indirect harmful effects on chickens such as decrease digestibility of fat, increase cell turnover rate, production of toxic metabolites from the protein fermentation and may also lead to poor growth performance.

### Beneficial roles of gut microbiota

Gut microbiota provides nutritional compounds to the host in the form of fermentation end-products and other secreted products such as SCFAs, specialized enzymes, amino acids, B and K vitamins and absorption of ions [7, 8, 31, 32]. Commensal bacteria generate SCFAs such as acetate, propionate, butyrate, and lactate in the GIT of chickens [19, 33]. These SCFAs have their specific role in the GIT such as contribution to energy by gluconeogenesis [34] and reducing undesirable bacterial species in the cecum [32]. SCFAs also stimulate gut epithelial cell proliferation, differentiation and increases the villus height, thereby increasing the absorptive surface area [34]. Acetate and propionate also act as an energy substrate for tissues. Recently, xylanase genes are isolated and overexpressed from the cecum of chickens which can degrade and digest complex substrate like non- starch polysaccharides which will encourage nutritionist and researchers to explore alternative feedstuffs to incorporate in large-scale industrial production [35].

Gut microbiota resists colonization of the chicken intestinal tract by pathogens and other non-indigenous microbes through competitive exclusion [7, 32, 36]. Attachment of non-pathogenic bacteria to the brush border of gut cell obstructs pathogens from attachment and entry into the cell. Indigenous microbiota of the gut suppresses the growth of pathogens by secreting organic acids and bacteriocins through direct stimulation of the immune system and compete for nutrition and attachment points to the mucosal wall [13]. In an in vitro experiment by Naidu et al. [37], *Lactobacillus* producing bacteriocin Reuterin was found effective in inhibiting the growth of *Salmonella, Shigella, Clostridium,* and *Listeria*. Increasing these types of useful bacteria along with substrates for their proliferation and metabolism improve feed intake and nutrient utilization by the host.

Experiments comparing conventionally reared versus germ-free animals show that commensal bacteria play a role in developing the intestinal host defenses, including the mucus layer, epithelial layer and lamina propria [8, 13, 32]. The mucus layer keeps both commensal and pathogenic microbes away from animal tissues. If the mucus layer is crossed, the epithelium acts as a barrier to enter inside the host tissue. The underlying lamina propria provides antibodies, cytotoxic and helper T cells, and sometimes also phagocytic cells. These immune cells not only combat pathogenic bacteria but also control the overcrowding of normal microbiota. Researchers also found that the antibody response in chicken is antigen-driven [38, 39]. Further evidence suggests that the intestinal immune system develops parallel to the development of gut microbiota. Thus, gut microbiota plays a significant role in maintaining immune homeostasis by preventing inflammation [40].

### Harmful roles of gut microbiota

Sometimes normal bacteria can have an adverse effect on gut health, even under ideal conditions. Commensal bacteria compete for nutrition with the host and produce toxic compounds as a byproduct of metabolism. The undigested protein of feed origin, true endogenous protein (mucin, epithelial cells, enzymes, and antibodies) and microbial proteins which bypass the small intestine and are available for the microbiota in the large intestine [41]. These microbiota ferment bypass proteins to produce toxic metabolites such as ammonia, amines, phenols, cresol and indoles which can impact intestinal cell turnover and even growth performance [41–43]. Also, any disorder to the epithelium of small intestine could lead to high protein level in the large intestine, resulting to increased protein fermentation and putrefaction as evidenced in a study [4]. In the study, birds challenged with *Eimeria maxima* showed elevation in biogenic amine level in the cecum, it might be due to disorder in integrity and absorptive capacity of the small intestine epithelium.

Despite several benefits to host, GIT microbiota can result in detrimental effects in certain special conditions. Intestinal microbes decrease fat digestibility by deconjugating bile acids [8, 42]. Bile acids and their salts are required to emulsify and absorb fat in the intestine. Catabolism of the bile salts in the gut by a variety of microbiota causes a decrease in lipid absorption and produces toxic products that inhibit the growth of chicken. Many authors have proposed that the reduction in amino acid catabolism and bile catabolism and increase in availability of nutrients are the primary physiology of how antibiotics improve animal performance [7, 44, 45]. Microbiota alters the intestinal morphology, cell turnover rates, and mucus secretion [8, 42]. Conventionally raised animals have higher small intestine weight due to its thicker walls, longer villi, and deeper crypts which allow infiltration of immune and connective tissue as compared to germ-free animals [46, 47]. It is also believed that an increase in thickness of the GIT wall and connective tissue decreases the nutrient uptake [7, 45]. Further, microbiota accelerates turnover rates of enterocytes and goblet cells such that the high cell turnover is accompanied by extremely high rates of metabolism and protein synthesis [48, 49]. This higher rates of metabolism and protein synthesis results in higher populations of immature cells that are less efficient in absorbing nutrients and are less able to provide efficient barrier due to having looser tight junctions [42].

As discussed above, microbiota plays a crucial role in host immunity development. However, there is inherent inefficiency when immune stimulation is maintained at a constant level, as appears to be the case in germ-free chickens, which contain lower serum Immunoglobulin G (IgG) compared to conventionally grown chickens [50]. Thus, microbiota uses explicitly IgA and IgG secretion which alone can cost several hundred grams of protein in a lifetime that is not directed towards the growth of chicken. According to Macpherson et al. [51], IgA is directed towards individually established gut flora and maintaining its population constant by controlling species entering from food and the environment. In poultry, gut metabolism accounts for 20–36% of the whole-body energy expenditure, primarily related to cell turnover required by microbiota [49]. Thus, the efficiency of nutrients from feed has to be reduced to achieve improved growth performance.

## Balance in gut ecology

The GIT of poultry harbors a complex and dynamic microbiome consists primarily of bacteria and low levels of protozoa, fungi, yeasts, bacteriophages, and viruses. These MCs intensively interact with the host and ingested feed. The composition of this microbiome is different in different parts of the GIT, with each section containing different niches. These MCs in different segments are affected by the flow of nutrients from the diet, the response by host immunity, other substances produced, and/or secreted making up this complex microbiome [9, 52]. Oviedo-Rondón et al. [9] suggested that the cross-talk between microbiota and host regulates the degree of immunity, symbiotic relationship, and production of endogenous proteins in response to pathogenic antigens. Even if there is an overall positive balance established between the microbiota and the host, still microbiota are classified into commensal and pathogenic organisms. Usually, pathogenic microorganisms are present in low concentration and can remain in the gut for more extended periods without any harmful effect to the host. Although some of the microbiota show beneficial roles in promoting a stable gut environment, they can act as pathogenic agents by producing toxic metabolites when the situation is unfavorable. Thus, a stable environment in the gut is a key to a healthy host. In addition, Oviedo-Rondón and Hume [53] explained the importance of maintaining the diversity of the gut MC, which in turn, improve gut health for better feed conversion and nutrient utilization in birds. A better understanding of this MC results in improvements in poultry health, productivity, and a reduction of food-borne pathogens, welfare, and overall environmental impact of poultry production for a more sustainable industry.

## Effects of gut microbiota on nutrient utilization, growth, and health

The GIT is the ultimate organ for host digestion and immunity and the proper functioning of this organ; gut microbiota should be in balance and dynamic state. Gut microbiota affects the metabolic processes directly such as *Clostridium cluster* XIV, and *Ruminococcus* can break cellulose and resistant starch [54]. Indirectly as most of the bacteria phylotypes abundant in higher AME utilizing and higher growth performing birds are firmly related to bacteria with known beneficial metabolic characteristics [55]. Also, the most dominant cecal microbes Firmicutes, and Bacteroidetes are correlated with body weight as their ratio is found significantly higher in obese hosts and lower in hosts of low to healthy body weight [56]. For proper intestinal function and integrity, bacterial fermentation plays an essential role by producing fermentation by-products such as SCFAs, especially butyrate, to provide energy to the epithelial cells and another SCFAs undergoes diffusion to enter different metabolic pathways. Other functions of SCFAs include regulation of intestinal blood flow, mucin production, enterocyte growth and proliferation, and intestinal immune responses [57]. *Lactobacillus* sp. is known to produce a variety of SCFAs and bacteriocins with bacteriostatic or bactericidal properties either by reducing pH or by modifying the receptors against pathogenic microbes [30].

## Modulating gut microbiota

Some feed ingredients and additives are reported to modulate gut microbiota and immune system of the host [1]. Antibiotics have been used to modify gut microbiota and were revered by farmers as they promote growth performance of poultry. However, concern about antibiotic resistance and other negative impacts of the use of antibiotics as a growth promoter, have forced poultry farmers to stop or limit their use in feed. Feed additives and supplements like probiotics, prebiotics, organic acids, and exogenous enzymes are used as an alternative to antibiotics to the modulate the gut microbiota with some success.

### Antibiotics

Antibiotics have been used for therapeutic and growth promoting prophylactic purposes in animals since the 1940s [58]. In a report by Oliver et al. [59], United States (US) alone uses about 24.6 million pounds of antibiotics annually, and most of these are used as a growth promoter rather than as a treatment of infections. Antibiotics are either synthetic drugs or are obtained from natural sources. These are used to kill or inhibit the growth of microorganisms in a broad sense, but these antibiotics also play some beneficial role in the gut. Early exposure of broiler chicken for short-term to the orally administered antibiotics (amoxicillin) has shown effects on the microbial colonization, mucosal gene expression and immune development in the later period up to 2 weeks post-hatch [17]. In a recent study by Wisselink et al. [60], adding antibiotics in drinking water changed the microbial community and immune parameters temporarily in the later phase of life (days 15 to 20). The dominant mechanism by which antibiotics work ranges from cell membrane destruction to reduction of growth-depressing metabolites produced by microbiota in the gut, especially ammonia and bile degradation products [61]. For the host, antibiotics have been shown to increase the nutrient availability in the gut that causes a decrease in amino acid catabolism and bile salts breakdown leading to an increase in the digestibility of dietary protein. Other beneficial effects of antibiotics include the efficient absorption of nutrients and nutrient utilization by the gut wall due to the thinner epithelium and decreased microbial use of nutrients; thus, more nutrients reach the host’s tissues [61]. Because antibiotics reduce the gut microbiota and their toxic metabolites, antibiotics have been widely incorporated into the poultry industry for decades. At the same time, irregular use and overuse of these antibiotics have been claimed to lead to the development of resistance by bacteria. This bacterial resistance causes a threat to the human and animal treatment as they transmit the genes for antibiotic resistance or may also exchange plasmid with inter or intra-species [62]. Their use as the prophylactic dose in animal feed has been banned in some jurisdictions like in one of the European Union (EC Regulation, No. 1831/2003) while other jurisdictions are considering or have imposed strict regulation on use or gradual ban in animal farming. This prohibition has already added pressure to the poultry farmers and nutritionists. For example, there is evidence that antibiotic growth promoters (AGPs) were useful in the prevention of necrotic enteritis in poultry, ban in use of AGP has led to increased incidence of necrotic enteritis cases. Antibiotics are also known for its anti-inflammatory role with the benefit of reducing waste of energy and utilizing in production [61]. Thus, there is an immediate need for identifying alternatives to antibiotics to maintain the balance of the ecosystem in the gut as well as to improve the overall performance of the birds [63].

### Probiotics

Probiotics also referred as direct-fed microbial (DFM), are single or mixed cultures of living non-pathogenic microorganisms, which, when administered in adequate amounts confers a health benefit on the host [64]. Bacterial species currently being used in probiotics are lactic acid bacteria (LAB), i.e., (*L. bulgaricus, L. acidophilus, L. casei, L. lactis, L. salivarius, L. plantarum*)*, Streptococcus thermophilus, Enterococcus faecium, E. faecalis, Bifidobacterium* sp. [65]. In addition to bacteria, fungi (*Aspergillus oryzae*) and yeast (*Saccharomyces cerevisiae*) are also used as probiotics [65]. Their mode of action involves multiple mechanisms, including competitive exclusion, promoting gut maturation and integrity, regulating the immune system, preventing inflammation, improving metabolism, improving growth, improve the fatty acid profile and oxidative stability in fresh meat [66], and neutralizing enterotoxins. Singh et al. [67] found increased apparent metabolizable energy and protein digestibility of fibrous diets in broiler chickens when supplemented with DFM along with multi-enzymes. However, some researchers did not found significant effect of single or multiple-strains DFM on growth performance of chickens [68]. Multiple-strains DFM showed better effects on local and systemic immune responses and competitive exclusion compared to single-strains DFM [68] Also, Kalia et al. [69] observed no differences in the growth performance of the RIR cross-bred chicken that was selected as the best performing breed out of all the breeds used in the trial. This could be due to the difference in the dose or an insufficient number of probiotic bacteria, nature and route of probiotics administrated, the difference in microbes among a range of altitudes, and the variation in the physiological state of the birds [63, 69]. Wang et al. [70] found that probiotics can improve gut microbiota diversity. Specifically, *Bacillus* sp. increased body weight and *Pediococcus pentosaceus* had higher average SCFAs content. They also identified that cecal microbiota, Bacteroidetes abundance was directly correlated with the content of propionate, butyrate, and isobutyrate, whereas the abundance of Firmicutes positively correlates acetate production in the cecum. Regarding immune responses, Brisbin et al. [71] reported that in the cecal tonsil cells of chicken, *L. acidophilus* induces T-helper cytokines whereas *L. salivarius* induces anti-inflammatory response more effectively. Also, the “Nurmi concept” is the most convenient example of an effective immune response by micro-organism whereby day-old chicks acquire enhanced protection against *Salmonella* infections when they are administered the complex microbiota of older chicks. In a study conducted by Cengiz et al. [72], no interaction was observed between stocking density and probiotic supplementation for performance, carcass yield, *Salmonella* and *Lactobacillus* population in the gut. Although, probiotics enhanced the performance during the starter phase only where high stocking density affected the birds negatively and stress indicators were not affected. Bai et al. [73] found that probiotics improve growth performance in early stage (1 to 21 d) of a chicken, but there was no dose response for 22–42 d when feeding 0.1% to 0.3%. As a result, the study recommended incorporating probiotics in 0.1% dose to chicks as an alternative to AGPs. Previously, Li et al. [74] also found that commercial probiotic mixture of yeasts and other microbes improve growth performance in starter age of broilers with no dose effect among 0.2% to 0.6%. Based on the reports available so far, probiotics in feed can be considered as one of the best alternatives to antibiotics in poultry diets to modulate gut microbiota as well as promote overall health and growth performance.

### Prebiotics

Prebiotics are non-digestible feed ingredients that are responsible for altering the composition and metabolism of gut microbiota selectively. Prebiotics has the ability to increase the number of bifidobacteria and other species that affect the health of host positively [63]. The β-glucan fed birds were found to have anti-*Salmonella* property by increasing the IgA-secreting cells, IgG level, and goblet cells causing immunomodulation to help birds boost immunity during *Salmonella* challenge [75]. Prebiotics also increase the number of the LAB in the gut that aid in the competitive exclusion of pathogens [76]. They also help to enhance defense mechanism. However, the mechanism by which they help in defense is not precise. It is supposed to increase the production of SCFAs leading to an acidic environment in the gut and suppresses pathogens, which also recover some lost energy from competition with bacteria [77]. According to Kim et al. [78], rapid clearance of pathogens because of prebiotic administration is a mechanism to boost immunity. In fact, prebiotics and probiotics have similar modes of action to maintain gut ecology and when provided in combination shows synergistic effect on the gut health [79]. Supplementation of slowly digestible prebiotics provides fermentable carbohydrates for microbiota in the distal large intestine, which in turn, suppress putrefaction. Owing to the supplementation of prebiotics in diet and its mechanism in the gut attributes to improvements in bird performance and energy utilization [27]. Though commonly used these days as an alternative to AGP, nature, characteristics, and type of prebiotic is crucial to understand as these variables influence the effects of the poultry. Commonly used prebiotics are oligosaccharides including inulin, fructooligosaccharides (FOS), mannanoligosaccharides (MOS), galactooligosaccharides, soya-oligxosaccharides, xylo-oligosaccharides, pyrodextrins, and lactulose. The FOS is the preferred substrate for bifidobacteria, helping it to bind to the host mucosa leading to the hindrance of pathogenic bacteria attaching to the gut mucosa, whereas MOS binds pathogens and excretes them with the digesta flow [63, 78]. Dietary supplementation with FOS also decreases *C. perfringens* and *E. coli* and increases *Lactobacillus* diversity in the chicken gut. The MOS also block binding of pathogenic bacteria notably *Salmonella typhimurium* to mannan receptors on the mucosal surface, thus prevent attachment or colonization [63]. Furthermore, developing method of a complete image of the GIT affected by pathogens using modern molecular techniques and bioinformatics pipeline will help understand the complex mode of action of prebiotics to control *Salmonella* [80]*.* Therefore, using preexisting prebiotics or developing new prebiotics can be a potential feed additive to replace AGP and modulate microbiota for better growth and improved health of poultry.

### Organic acids

Organic acids are the normal constituents of the plant and animal tissues. Previously organic acids were used as a preservative to prevent deterioration and increase shelf-life of perishable food pre-harvest and post-harvest as it controls the microbial contamination [81]. It includes acids such as lactate, acetate, propionate, butyrate, tannic, fumaric, and caprylic acids, among others. These acids play a beneficial role in the gut health and performance of birds [82]. Saki et al. [82] found that organic acid increases the LAB count in the ileum and cecum of broiler chickens. The organic acid is also produced in the host gut after fermentation of carbohydrates, especially in the ceca of birds where the microbial population and diversity is at its highest level [63]. Each of these acids is utilized in different ways in the body of the host. Acetate is carried to the liver as an energy substrate for muscle tissue. Propionate is converted to glucose in the liver by the process of gluconeogenesis. Butyrate in small intestine enterocytes helps in the proliferation, development and serves as a vital source of energy for host metabolic activities [1]. However, butyrate does not always show positive effects, which largely depends on its location and concentration in the GIT [83]. The organic acids lower chyme pH which increases pepsin activity. The peptides arising from pepsin proteolysis trigger the release of hormones gastrin and cholecystokinin, which also helps to improve growth as this may increase protein digestion [84]. The mechanism of action could result in improved body weight gain and feed conversion ratio and decreased cumulative feed consumption [85], suppressing bacterial cell enzymes [63], and reduced pathogens like Enterobacteriaceae and *Salmonella* [82]*.* Supplementation of organic acids may affect cell membrane or cell macromolecules or interfere with nutrient transport and energy metabolism leading to the death of bacteria [81]. The effectiveness of these compounds as antimicrobial agents in the gut depends on the ability of acids to change from the un-dissociated to the dissociated form, the p*K*a value, and its hydrophobicity. Supplementation of these acids should be done in proper dose otherwise it will lead to depressed villus height and width, as well as crypt depth [86]. Thus, organic acids have been incorporated in feed or in water to affect positively on the prevention of GIT diseases, immunity, nutrient digestibility, and overall growth performance of the broiler chickens.

### Exogenous enzymes

Enzymes are specialized proteins that catalyze or accelerate the chemical reaction. The enzyme activity may be substrate dependent or through the particular site on substrates such as fat, protein, or carbohydrate. Commonly used exogenous enzymes in poultry diets are β-glucanase, xylanase, amylase, α-galactosidase, protease, lipase, and phytase [87]. The role of exogenous enzymes is to fulfill the absence of endogenous enzymes, to counter the anti-nutritional factors present in conventional and unconventional poultry diet. These exogenous enzymes, in combination with non-conventional ingredients, are used to reduce the cost of feeding and to utilize the non-conventional feed ingredients efficiently [88] as non-conventional feedstuffs are typically rich in fibers [1] and are not utilized by endogenous enzymes of poultry. Also, a portion of starch and protein of these non-conventional feedstuffs are entrapped in the fiber matrix, making it unavailable for endogenous enzymes of animals, but these nutrients can be made available for utilization by use of exogenous enzymes [89]. Accordingly, NSP degrading enzymes which produce oligosaccharides could also reduce the putrefaction in the cecum as bacteria prefer carbohydrate as a substrate for fermentation when both carbohydrate and protein are available in the gut [41].

Enzyme supplementation is also essential for environmental issues such as pollution of soil and water with nutrients, pathogens, fouling of environment and heavy metals which occurs due to poor excreta management, as it may reduce the pollutant potential of excreta [88]. Carbohydrase supplementation increases the proportion of lactic and organic acids, reduced ammonia production, and increased SCFA concentration which is indicative of hydrolysis fragmentation of NSP and supporting the growth of beneficial bacteria. Supplementation of multienzyme (xylanase, amylase, and protease) optimized the utilization of fibers, leading to better growth performance of broiler chicken [90]. In an experiment with barley-based diet, β-glucanase supplementation decreased ileal viscosity and affected SCFA concentration in the crop and ceca due to the shift in resident microbial activity. The role of β-glucanase in other segments of the GIT is unknown [91]. When exogenous enzymes were supplemented to degrade NSP in a barley-based diet, gut microbial communities varied significantly among gut sections except between the duodenum and jejunum [92]. Exogenous enzymes are also beneficial to control salmonella that is transferred horizontally. The efficiency of these exogenous enzymes depends upon the diet composition, animal strain, sex and age, and digesta flow rate also the type of enzyme supplemented [87, 93]. Yang et al. [93] reported the growth-promoting effects of enzymes linking it to the mucosal morphology of the small intestine. They also stated that the crypt depth of the jejunum was reduced along with an increase in the membrane enzyme activity and role in the last step of digestion causing the improved growth of chicken by supplementing xylanase in diets. Also, Cowieson et al. [94] noted the beneficial role of exogenous protease by decreasing undigested protein from diet or endogenously produced to reach the caudal gut reducing inflammation and maintaining tight junction integrity. Exogenous enzymes are multifactorial in action due to its role in the partitioning of nutrients and help in the growth of specific microbiota by producing nutrients for them [95]. These enzymes are being used as an integrated solution to reduce the economic burden not just by limiting GIT pathogens but also by reducing medication costs, variability in animal performance, and reducing mortality by improving the gut health [96]. Although the exogenous enzyme has many benefits to the poultry, there are still some limitations imposed to health condition, disease challenge, quality of feed, pH and digesta retention time in the GIT [97]. Therefore, nutritional strategies to overcome limitations could help in effective utilization of unconventional feed ingredients to produce cost-effective feed for broiler chickens.

## Conclusion

To achieve optimal microbiota for better growth and improved health of poultry and to develop cost-effective feeding program, there is a need to manipulate gut microbiota through strategies such as the use of feed additives supplements singly or in combination in diets. Previously, antibiotics growth promoters were most commonly used to manipulate gut microbiota. Due to concern over the use of in-feed antibiotics, alternatives are being explored and applied. As alternatives, several feed additives including probiotics, prebiotics, organic acids, and exogenous enzymes are available and have been successfully used for modulating gut microbiota for better health and efficient production of poultry. Though recognized as a forgotten organ, gut microbiota is an essential component of intestinal ecology. A better understanding of gut microbiota and its interaction or balance with other organisms is crucial in understanding the composition of gut ecology, the effect of feed supplements on the modulation of gut microbiota, and finally, the beneficial and harmful effects of the microbiota. However, advanced techniques have only evolved in recent years. Therefore, there is only limited evidence available on how specific dietary components affect the gut microbiota. The main sites of bacterial activity are the crop and the ceca and to the lesser extent, the small intestine. These bacteria produce various metabolites using diets that can be beneficial or harmful to the host. Role of microbiota on the physiological, developmental, nutritional, and immunological processes of the host, leads to a beneficial effect on host gut health, performance and well-being of poultry birds in a range of aspects. Beneficial bacteria can protect the host from pathogenic bacteria by the different competitive mechanism. These bacteria are also involved in the development of the intestinal immune system. Microbiota can be a significant hindrance to growth performance due to enormous losses of proteins and high expenditure of metabolic energy. They can also have a negative impact on vitamin nutrition. Thus, modulating gut microbiota is very important in the post-antibiotic era. As reviewed in this paper, alternatives to antibiotics such as probiotics, prebiotics, organic acids, and exogenous enzyme tend to modulate gut microbiota. After in-depth understanding of the role of these dietary supplements on the overall performance of poultry, the next steps would be to identify alternative sources (plant, animal or other origins) rich in these supplements. Moreover, studies focused on the combination of these feed additives for their synergistic and agonistic approach may contribute to filling the gap of information on their combined effects.

## Abbreviations

AGP: Antibiotic growth promoter
CFU: Colony forming unit
DFM: Direct-fed microbial
FOS: Fructooligosaccharides
GIT: Gastro-intestinal tract
IgG: Immunoglobulin G
LAB: Lactic acid bacteria
MC: Microbial community
MOS: Mannanoligosaccharides
RDP: Ribosomal database project
SBM: Soybean meal
SCFA: Short chain fatty acid
TRFLP: Terminal restriction fragment length polymorphism
US: United States
WS-NSP: Water-soluble non-starch polysaccharides
